# Invisible Victims: Delayed Onset Depression among Adults with Same-Sex Parents

**DOI:** 10.1155/2016/2410392

**Published:** 2016-05-29

**Authors:** D. Paul Sullins

**Affiliations:** Department of Sociology, The Catholic University of America, Washington, DC 20064, USA

## Abstract

The relationship of elevated depression risk recently discovered among adult persons raised by same-sex parents with possible precipitating conditions in childhood has not previously been acknowledged. This study tests whether such inattention is supportable. Logistic regression based risk ratios were estimated from longitudinal measures of mental health outcomes observed in three waves (at ages 15, 22, and 28) of the US National Survey of Adolescent to Adult Health (*n* = 15,701). At age 28, the adults raised by same-sex parents were at over twice the risk of depression (CES-D: risk ratio 2.6, 95% CI 1.4–4.6) as persons raised by man-woman parents. These findings should be interpreted with caution. Elevated risk was associated with imbalanced parental closeness and parental child abuse in family of origin; depression, suicidality, and anxiety at age 15; and stigma and obesity. More research and policy attention to potentially problematic conditions for children with same-sex parents appears warranted.

## 1. Background

In research and policy settings, children in unique distress with same-sex parents are not supposed to exist. Most studies have reported “no differences” in well-being, most often using psychometric measures of depression or anxiety, supporting a lapse in policy attention to the potential needs of such children. Uniformly benign findings for this population have recently been challenged, however, by several original research efforts [1–3], the rediscovery of older studies [4, 5], and the reanalysis of studies long thought to support “no differences” [6].

The sparse and gendered nature of the same-sex parent population largely restricts research in this area to the examination of small samples of lesbian parents. Unfortunately, this difficulty has prompted an almost universal dependence on convenience samples [7, 8] recruited, with knowledge of study goals, from internet surveys, “LGBT events, bookstore and newspaper advertisements, word of mouth, networking, and youth groups” [2]. Reanalyses have confirmed, not surprisingly, the presence in such samples of strong ascertainment bias, social desirability bias, and/or positive reporting bias [9–11]. In most studies, lack of statistical significance using simple bivariate tests in such samples is then erroneously interpreted as strong evidence of “no differences” in the population, even when difference in estimates or effect sizes are substantively large and even though the sample is not representative [12].

In fact, only four of the several dozen studies alleging “no differences” have examined a representative sample. The largest and most recent of these, Rosenfeld's analysis of 3,174 same-sex parented children on the US Census, is discussed in Section 5. The other three are related studies based on a single sample, a group of 44 adolescents with lesbian parents captured on over 20,000 population-representative cases of the initial wave of the National Longitudinal Survey of Adolescent Health (“Add Health”) [13–15]. Sullins, however, recently found that most (27 of the 44) adolescents in this sample allegedly with same-sex parents were actually living with opposite-sex parents including, for most of them, their biological father as well as their mother. After removing the mixed cases, the remaining sample members fared significantly worse on psychometric measures of anxiety and autonomy than did their adolescent counterparts with opposite-sex parents, albeit comprising only 17 cases [16]. Other studies employing large representative samples have also found higher depressive symptoms, indicated by the Center for Epidemiological Studies Depression Scale (CES-D [17]), among younger same-sex parented children [18] and adults who report having had a same-sex-related parent at some point during childhood [1]. The design and methodology of Regnerus' study were the subject of a brief but vigorous debate [19–21], which turned largely on definitional issues [22].

No study has yet explored the connection, if any, between late onset distress and precipitating conditions in children in this population, and no research reporting “no differences” has yet investigated parental child abuse or adult onset difficulties [24]. The present study aims to amend these gaps in the research. It improves on the sample limitations of prior studies by employing data that are both representative and longitudinal, following the corrected Add Health sample of adolescents with lesbian parents, the most well-regarded small sample used in this field to date, through Wave IV, thirteen years after the initial interview at age 15 (on average). It improves on prior methods by the use of standard psychometric scales, to the extent possible, and the estimation of relative risk by logistic regression models with appropriate survey weighting. As the first study to examine children raised by same-sex parents into early adulthood, this exploratory study aims to contribute new information for understanding of the effects of same-sex parenting through the life-course transition into early adulthood.

The analysis followed a grounded theory approach, first identifying the presence or absence of pertinent differences by family type and then developing and testing grounded hypotheses, drawing both from the observed bivariate characteristics of the data and prior research where applicable. For clarity the research presentation will also follow this order, with the formulation of hypotheses presented following initial bivariate results.

## 2. Data and Methods

This study implements data from the National Longitudinal Study of Adolescent to Adult Health (“Add Health”), which has followed a representative sample of American adolescents through interviews at average age 15 (Wave I, baseline) in 1995, age 22 in 2002 (Wave III), and age 28 in 2008 (Wave IV, terminus). Wave I completed home interviews with 20,745 American adolescents aged 13–19 and their mothers selected from a sample of US high schools and rendered representative through the application of poststratification weights, reduced by mortality to 15,701 at Wave IV. Missing data on analytical control variables reduced available cases in the present analysis to 12,288, which was reduced further by missing data on variables of interest, depending on the analysis, to as low as 8,762.

Same-sex parents were identified using the procedure described by Wainright et al. [13] as corrected by Sullins [6]. To assure accurate identification of same-sex partners, sex of partner was independently identified by both mother and respondent. All analyses were performed with Stata 13 statistical software (StataCorp LP), incorporating the design features of the survey following guidelines published by the Carolina Population Center, University of North Carolina [25]. Procedures for data access and analysis were approved by the institutional review board of the Catholic University of America and in agreement with the sensitive data security plan approved by Add Health.

The comparison sample comprised 20 unweighted cases of adolescents with same-sex parents, consisting of 17 lesbian partners and 3 gay male partners. Analytical controls adjusted for demographic differences between the general population and this group of adolescents, which was older (15.4 years, SE .97 versus 15.0 years, SE .12) and disproportionately female (72.1%, SE 11.4 versus 49.0%, SE .55) and white (81.1%, SE 10.6 versus 73.2%, SE 2.5). Compared to the general parent population, the same-sex parents were much more highly educated (66.2%, SE 15.6 had a college degree, versus 26.1%, SE 1.5 of parents overall) but had lower income ($36.5k, SE $6k versus $45.2k, SE $1.7k in 1995). These differences are consistent with prior studies of lesbian parents, which have found that, despite possible higher education, the combination of two female incomes and a higher proportion of household caregiving generally results in lower income [26, 27].

### 2.1. Variables in the Analysis

Table 1 presents for reference the adjusted means by family type for the variables in the analysis. Depressive symptoms at Wave I and Wave IV were measured by the Center for Epidemiologic Studies' Depression Scale (CES-D) which was administered in the in-home interview [17]. The items in the scale name a list of symptoms such as feeling sad, lonely, tired, or bothered about things. The response range for each item is from 0 (never or rarely) to 3 (most of the time or all of the time). The Wave IV interview employed an abbreviated version that included 5 of the 19 items and identified depressed individuals by means of an item-weighted classification performed by Add Health analysts at the University of North Carolina.

Anxiety at Wave I was assessed by a series of six items which asked about both physical conditions such as sleeplessness or poor appetite and more direct indicators of emotional distress such as moodiness, fearfulness, or frequent crying. Parental distance was constructed from two variables asking how close the respondent felt to his or her father and mother. Respondents reporting that they felt very little or no closeness to one or both parents were coded as experiencing parental distance. Perceived stigmatization was measured by two questions that asked about being treated with less respect than other people and feeling as good as other people. Respondents scoring high on the first item and low on the second one were classified as having experienced or internalized stigma. Body mass index (BMI) was computed from physical measurements of height and weight at the Wave IV interview. BMI over 30 was classified as obese. Women currently pregnant were excluded.

Retrospective questions at Waves III and IV asked about adult mistreatment during childhood, including whether a parent or caregiver had “slapped, hit or kicked you,” said “things that hurt your feelings or made you feel you were not wanted or loved,” or “touched you in a sexual way, forced you to touch him or her in a sexual way, or forced you to have sex relations.” Respondents reporting any physical, verbal, or sexual abuse at either Wave were coded positive for abuse victimization. Four-fifths (79%, 95% CI 77–80) of reported mistreatment was verbal abuse.

Adjustments for sociodemographic differences included parent education and income, respondent sex, age, and race, and respondent education and household income at Wave IV. Race or ethnicity was measured as five categories modeled on the US Census: Hispanic, white, black, Asian, and other. Sex was measured as male or female and age in years. Parental income in dollars was reported at Wave I; respondent income at Wave IV was measured in eleven categories with midpoints from $7,500 to $150,000. Education was expressed in four categories: no high diploma, high school diploma only, college degree, or more. Risk of depression declined moderately with higher income and higher education.

## 3. Hypotheses

Emergent differential depression risk for the children with same-sex parents may be related to several conditions or factors in their unique circumstances. The following discussion in this section reflects four associations reflecting initial analysis and suggestions from prior literature. Since they are constrained by available data, they are neither mutually exclusive nor comprehensive; all may have some effect, as well as other, unobserved, associations.

### 3.1. Mental Health at Baseline

Abundant research has found that depression in adolescence is one of the largest predictors of depression in adulthood. In the present study, adolescent mental health also serves as a direct test of the idea that differential risk by family type is related to emergent adulthood, since that idea must be rejected or qualified to the extent that mental health conditions in adulthood are associated with the same conditions at adolescence.

### 3.2. Family of Origin Factors

Two characteristics that differed significantly by family type, level of parental abuse and the pattern of parent-child closeness, have previously been implicated in the onset of adult depression. The link between adult depression and reported childhood sexual, physical, or verbal abuse is well known [28, 29]. Recent studies have argued that verbal abuse may be more strongly related to negative internalization and the development of depression than physical or sexual abuse [30]. Parent-child closeness has also long been linked to emotional development in adolescence and early adulthood [31, 32]. Absence of the father during adolescence—the most common form of deficient parental closeness—has been associated with a variety of negative outcomes including depression [33]. Amato and Afifi found that an imbalance in current parental closeness, that is, being close to one parent but not the other, also lowered subjective well-being for adult children in their twenties [34]. In the present study, preliminary modeling on closeness to mother and father confirmed that the bulk of the variation in depression was related to whether the respondent had been (at Wave I) or was currently (at Wave IV) close to both parents or not.

### 3.3. Wave IV Association Pathways

Differential social stigma or sensitivity to stigma faced by same-sex families has often been cited as an impediment to psychosocial development in children with same-sex parents, including adult children raised by lesbians [35, 36]. In the present data such persons were almost five times more likely to have experienced stigma or perceived stigma as adults, suggesting that their higher mental distress may be associated with a strong differential experience of stigma.

Obesity was also much higher among the adults with same-sex parents and has in turn been linked to higher depression. In a 2010 meta-analysis summarizing 15 longitudinal studies, Luppino and colleagues reported that obese Americans were twice as likely (OR 2.1, 95% CI 1.5–3.0) to develop depression as were nonobese Americans [37]. Obesity has also been found to be linked to childhood abuse [38] and to stigma that can contribute to depression [39].

## 4. Results

### 4.1. Bivariate Results

The initial evidence for delayed onset of higher depression risk for the same-sex parented children is presented in Table 2, illustrating comparisons from Table 1. Depression risk for this group at Wave I was lower than for the general population, after accounting for differences in family socioeconomic status, but by Wave IV the risk ratio for depression had increased to 2.6. Most of the increased risk was due to a marked increase in the rate of depression for the children with same-sex parents, from less than a fifth at Wave I to over half at Wave IV, corresponding to a slight decrease in depression among the general population over the same period of maturation. By Wave IV the difference is both statistically and substantively significant, with an effect size approaching a full standard deviation.

The dependent variables in the analysis, already presented in Table 1 and discussed in Section 3, were selected from a large number of possibilities by retaining those that met at least one of two tests: the condition either was plausibly associated with affectivity or showed a similar pattern of increase or persistence over the transition into adulthood as did the independent variable of interest, that is, the depression risk ratio.

Three possible depression covariates demonstrated a strong risk ratio at Wave I which they retained at Wave IV: suicidality, parental distance, and obesity. The risk ratio for suicidality, a mental health measure directly related to affective distress, was, like depression, higher at Wave IV than at Wave I. At both Wave I and Wave IV, more of the adolescents with lesbian parents reported feeling close to their mother, while fewer reported feeling close to their father, compared to the case for the comparison group. All of the former group at Wave I and almost all of them at Wave IV felt extremely or very close to their mother; less than half at both points in time reported feeling extremely or very close to their father.

Obesity was much more common among the same-sex parented adults than for the comparison group and increased substantially among both groups into early adulthood. By Wave IV, over two-thirds (72%) of the same-sex parented adults were measured as obese, compared to 37% of the comparison group. Anxiety, a direct measure of affectivity, was also higher at Wave I and perceived stigma was higher at Wave IV; unfortunately these variables were not measured at other waves.

### 4.2. Multivariate Model

Table 3 presents logistic regression results modeling the association of the hypotheses outlined in Section 3 with the depression risk ratio for same-sex parented adults at Wave IV. Model 3.1 estimates that the children with same-sex parents are 2.6 times more likely to experience depression at Wave IV, after adjusting for demographic differences. This model is identical to the corresponding estimates presented in Tables 1 and 2.

In interpreting the models in Table 3, it is important to bear in mind that the outcome of interest is not depression risk but the* differential* risk of depression between the same-sex parented children and the comparison group, as expressed by the risk ratio. As hypothesized, mental health measures of depression, suicidality, and anxiety at adolescence (Wave I) were strong predictors of mental health outcomes in early adulthood (Wave IV). However, as Model 3.2 shows, they were associated with only a moderate (13%) reduction in risk ratio at Wave IV, consistent with the mixed results for mental health differences for the same-sex parented children at Wave I. Likewise, child abuse and lack of closeness to parents, both strongly associated with depression and more prevalent with same-sex parents, were associated with only another 13% reduction in the estimated risk ratio, from 2.25 to 1.95 in Model 3.3. Perceived stigma, the most powerful single predictor of depression at Wave IV in the analysis, was associated with only a further 9% moderation of the risk ratio. Altogether, Model 3.4, which included mental health at adolescence, family of origin factors, and perceived stigma, reduced the risk ratio toward unity by about half (from 2.58 to 1.77). The six independent variables in this model all are strongly associated with depression risk but appear to affect both groups of children more similarly than differently.

By contrast, obesity at Wave IV was not a significant predictor of depression risk itself but had a very strong association with the increased risk of depression for the children with same-sex parents. The odds on depression associated with obesity, shown in Model 3.5, are not significantly or substantively different from unity. However, including obesity in the model, in addition to the other predictors discussed, accounts for all of the remaining difference in risk between the two groups of children. Obesity thus has no direct association with depression risk itself but has the strongest association of any variable in the analysis with the higher risk ratio for depression for the children with same-sex parents.

## 5. Discussion

The presence of delayed onset depression for children with same-sex parents may help to explain both findings of “no difference” in mental health at adolescence and substantial differences in adult mental health when compared to children with opposite-sex parents.

Interpretation of these limited small-sample findings is necessarily speculative. Emergent differences in depression risk associated with obesity, parental distance, and suffering abuse may suggest an association of emergent depression risk with maturation or family formation processes following departure from the family of origin. The modal case for females with same-sex parents in these data was to depart the family of origin for a male sexual relationship in their teens, which may favor an explanation related to family formation. As noted above, 72% of the same-sex parented children were female; the estimated Wave IV risk ratio for depression for these cases is higher, at 2.8 (95% CI 1.9–4.1), than for the sample as a whole.

Greater closeness to one or both same-sex parents, compared to children with heterosexual parents, has often been documented in the study population [40] and is confirmed by the present study. However, no study has yet examined the effect of the corresponding distance from the excluded biological parent. As Table 1 shows, the very high rate of parental distance at adolescence (93%) for this population moderates by early adulthood, while increasing somewhat for the general population, suggesting the partial resolution of any differential distress related directly to this factor. On the other hand, increased distress in early adulthood may be related to conflicts or ambivalence involving resolving parental distance while at the same time establishing independence from the family of origin.

The high prevalence of obesity in the adult children of lesbian parents also reflects known characteristics of this parent population. Fredriksen-Goldsen and colleagues found that obesity among lesbians was associated with a lower health-related quality of life, which predicted higher mental distress among both lesbian and (nonobese) heterosexual women [41]. On the other hand, Cochran and Mays, comparing samples of heterosexual and lesbian women, found that mental distress among lesbians was not significantly higher after controlling for lower physical health [42].

The well-documented tendency toward greater levels of intimate violence in same-sex partnerships [43, 44] appears also to be present in parental relationships. Prevalence was notably high. Ninety percent of the same-sex parented children reported parental abuse at Wave III, dropping only to 85% at Wave IV. Prior studies have not documented this abuse, most likely because almost all have been based on parental reports, which minimize abuse self-reporting, and none (to my knowledge) have ever asked directly about parental abuse. In the only prior study based on retrospective reports, children with lesbian mothers (including those temporarily in a lesbian relationship) reported a substantially higher rate of sexual abuse, at 23%, than did those with consistently heterosexual parents [1].

The results of the present study may be inconsistent with those of a 2010 study by Rosenfeld which found no significant difference in school completion rates for a sample of 3,174 children with same-sex parents drawn from the 2000 Census Public Use Microsample [45]. This study is the only large representative-sample study of same-sex parents to date that has found “no differences.” However, Rosenfeld ignored (or was unaware of) a misclassification error affecting the attribution of same-sex couples on the 2000 Census reported earlier by Black and colleagues [46]. After reviewing the census attribution procedures, Black et al. concluded that at least forty percent of the cases in the same-sex couples sample “are actually different-sex married couples” [46, page 9], warning researchers that “many of the inferences drawn from these data are incorrect” [46, page 10]. Allen failed to replicate Rosenfeld's finding using the Canadian census [2] and has disputed Rosenfeld's analysis [47]. Even if Rosenfeld's finding is correct, however, it is not necessarily inconsistent with the present findings. Sullins, using the same sample as the present study, found that the children with lesbian parents attained higher grade point averages in high school while at the same time displaying higher affective distress [6]. Sullins speculated, on the basis of retrospective narrative reports from children raised by lesbians, that high control parental behavior may account for this apparent anomaly. The relationship between school performance and affectivity for same-sex parented children may be different compared to that in the general population, may vary by parental gender, and merits further focused research to clarify these disparate findings.

*Limitations.* Despite the signal strengths of Add Health as a large nationally representative longitudinal dataset and notwithstanding the strong significance for contrast effects reported above, the very small size of the sample of children raised by lesbians imposes important limits and prompts great caution regarding the conclusions of this study. As with all observational studies, causal inference is not possible. Moreover, many subtle distinctions and pathways of influence simply cannot be addressed with only 20 cases, and unobserved differences between the parent comparison groups may well confound some or all of the child differences observed. In particular, the lack of useful measures for parent mental distress, depression, family history of violence, alcohol consumption, and substance abuse precluded examination of important familial risk factors which may be associated with child distress. For these reasons, the findings of this study should be considered only provisional and exploratory until and unless they are confirmed by further research.

## 6. Conclusion

The emergence of higher depression risk in early adulthood, coupled with a more frequent history of abuse victimization, parental distance, and obesity, suggests that the inattention of research and policy to the problems of children with same-sex parents is unwarranted. As initial results, the present findings should be interpreted with caution and balance, based on the limited evidence presented, and (it is hoped) neither exaggerated nor dismissed out of hand on preconceived ideological grounds. However, well-intentioned concern for revealing negative information about a stigmatized minority does not justify leaving children without support in an environment that may be problematic or dangerous for their dignity and security.

## Figures and Tables

**Table 1:** Adjusted means by family type for variables in the analysis, showing risk ratio, significance, and effect size: Add Health Waves I–IV.

	OS parents	SS parents	Risk ratio	95% CI	*p* > ln(OS/SS) = 0	Effect size (SMD)
	% (SE)	% (SE)				
Measured at Wave I (baseline)						
Depressed (CES-D)	21.8 (.66)	18.3 (14.4)	.83	.2–3.9	.82	−.13
Suicide ideation	13.6 (.44)	43.5 (19.0)	3.2	1.4–7.5	.007	.89
Anxiety	56.9 (.91)	89.9 (7.8)	1.6	1.3–1.9	.000	1.09
Distant from one or both parents	35.8 (.90)	93.2 (7.5)	2.6	2.2–3.1	.000	1.83
Obese	13.8 (.60)	30.8 (22.3)	2.2	.54–9.1	.27	.58
Measured at Wave III (*t* + 6)						
Child abuse by parent (verbal, physical, and sexual)	58.2 (.01)	92.0 (6.2)	1.6	1.4–1.8	.000	1.17
Measured at Wave IV (*t* + 13)						
Depressed (CES-D)	19.7 (.63)	51.0 (15.1)	2.6	1.4–4.6	.001	.85
Suicide ideation	7.1 (.43)	30.1 (21.4)	4.2	1.0–17.1	.04	.97
Perceived stigma	7.0 (.36)	36.7 (24.6)	5.2	1.4–19.4	.01	1.17
Distant from one or both parents	43.5 (.81)	72.6 (14.6)	1.7	1.1–2.5	.01	.71
Obese	37.2 (.81)	71.9 (17.8)	1.9	1.2–3.2	.009	.84
*N*	12,268	20				

Table values show logit estimates adjusted for parents' education and income and respondent's age, sex, race, education (at Wave IV), and income (at Wave IV). Test shown tests the equivalence of the logit coefficients, that is, ln(OS/SS) = 0. Effect size reports standard mean difference following Chinn [23].

**Table 2:** Analysis showing late onset depression: Add Health Waves I–IV.

	OS parents	SS parents	Risk ratio	95% CI	*p* > ln(OS/SS) = 0	Effect size (SMD)
	% (SE)	% (SE)				
Depression (CES-D)						
Wave I	21.8 (.66)	18.3 (14.4)	.83	.2–3.9	.82	−.13
Wave IV	19.7 (.63)	51.0 (15.1)	2.6	1.4–4.6	.001	.85
*Change Wave I-Wave IV*	−2.1	+32.7	3.1	—	—	+.98
*N*	12,268	20				

Table values show logit estimates adjusted for parents' education and income and respondent's age, sex, race, education (at Wave IV), and income (at Wave IV). Test shown tests the equivalence of the logit coefficients, that is, ln(OS/SS) = 0. Effect size reports standard mean difference following Chinn [23].

**Table 3:** Logistic regression models predicting risk ratio for depression at Wave IV by origin family type (same-sex parents versus opposite-sex parents): Add Health Waves I–IV.

	Model 3.1	Model 3.2	Model 3.3	Model 3.4	Model 3.5
Risk ratio (SS/OS)	2.58^*∗∗∗*^ (1.4–4.6)	2.25^*∗∗∗*^ (1.2–4.1)	1.95^*∗∗*^ (1.0–3.7)	1.77^*∗*^ (.93–3.4)	.99 (.37–2.7)
Effect size (SS/OS)			.56	.50	.00
Wave I mental health					
Depression		1.66^*∗∗∗∗*^	1.57^*∗∗∗∗*^	1.44^*∗∗∗∗*^	1.47^*∗∗∗∗*^
Suicide ideation		1.86^*∗∗∗∗*^	1.71^*∗∗∗∗*^	1.70^*∗∗∗∗*^	1.61^*∗∗∗∗*^
Anxiety		1.43^*∗∗∗∗*^	1.35^*∗∗∗*^	1.36^*∗∗∗∗*^	1.34^**∗∗∗**^
Family of origin factors					
Parental child abuse			1.86^*∗∗∗∗*^	1.77^*∗∗∗∗*^	1.72^*∗∗∗∗*^
Parental distance			1.51^*∗∗∗∗*^	1.46^*∗∗∗∗*^	1.44^*∗∗∗∗*^
Perceived stigma				3.55^*∗∗∗∗*^	3.54^*∗∗∗∗*^
Obesity (Wave IV)					1.05
*N* (unweighted)	15,698	10,459	8,762	8,762	8,762

Numbers in parentheses report the 95% confidence interval. Significance tests equality of coefficients, that is, ln(OS/SS) = 0. ^*∗*^ *p* < 0.10; ^*∗∗*^ *p* < 0.05; ^*∗∗∗*^ *p* < 0.01; ^*∗∗∗∗*^ *p* < 0.001. Significant results are in boldface. All models include adjustments for demographic control variables: age, sex, race, education (at Wave IV), income (at Wave IV), and parent education and income. Coefficients report odds ratios except as noted.

## Data Access

Information on how to obtain the Add Health data files is available on the Add Health website (http://www.cpc.unc.edu/addhealth).
