# Expression of Concern on “Invisible Victims: Delayed Onset Depression among Adults with Same-Sex Parents”

**DOI:** 10.1155/2017/4981984

**Published:** 2017-09-05

**Authors:** Depression Research and Treatment

On behalf of Hindawi Limited, the publisher of* Depression Research and Treatment*, we would like to express our concern with the article titled “Invisible Victims: Delayed Onset Depression among Adults with Same-Sex Parents” published in* Depression Research and Treatment* in 2016 [1].

The article has been cited to support arguments about same-sex marriage that Hindawi believes to be hateful and wrong. These arguments do not represent the views of Hindawi, our staff, or the editorial board of* Depression Research and Treatment*. We strongly condemn any attempt to justify hate speech or bigotry through reference to the scholarly record.

In June 2016, several readers raised concerns about this article. At that time, we evaluated the article's peer review process and brought several concerns to the handling editor's attention. These included: the study's small sample of same-sex parents, the lack of discussion of other influences such as family breakup on the wellbeing of the children included in the study, the implied causation in the title “Invisible Victims,” and the potential conflict of interest implied by the author's position as a Catholic priest.

The handling editor believed the article's reviewers addressed these concerns, and the author made sufficient revisions to the article to address these flaws. In the editor's opinion, the limitations of the study did not warrant further correction or retraction. As publisher, Hindawi does not overrule the editorial decisions of our academic editors in such cases.

Nevertheless, Hindawi felt it was important for the criticisms of this study to become part of the scientific record. We invited Dr. Nathaniel Frank, a critic of the article and director of the “What We Know” project (http://whatweknow.law.columbia.edu/) at Columbia Law School, to publish a letter to the editor in* Depression Research and Treatment* making these concerns visible to the journal's readers [2]. That letter is available at https://dx.doi.org/10.1155/2016/3185067. We also published a subsequent response from Dr. Sullins [3].
